# Magnetic Polyurea Nano-Capsules Synthesized via Interfacial Polymerization in Inverse Nano-Emulsion

**DOI:** 10.3390/molecules24142663

**Published:** 2019-07-23

**Authors:** Suzana Natour, Anat Levi-Zada, Raed Abu-Reziq

**Affiliations:** 1 Institute of Chemistry, Casali Centre of Applied Chemistry and Centre for Nanoscience and Nanotechnology, The Hebrew University of Jerusalem, Jerusalem 9190401, Israel; 2 Department of Entomology-Chemistry, Agricultural Research Organization, Volcani Centre, Rishon Lezion 7505101, Israel

**Keywords:** polyurea nano-capsules, magnetic nanoparticles, nano-emulsions, interfacial polymerization, composite nanomaterials

## Abstract

Polyurea (PU) nano-capsules have received voluminous interest in various fields due to their biocompatibility, high mechanical properties, and surface functionality. By incorporating magnetic nanoparticle (MNPs) into the polyurea system, the attributes of both PU and MNPs can be combined. In this work, we describe a facile and quick method for preparing magnetic polyurea nano-capsules. Encapsulation of ionic liquid-modified magnetite nanoparticles (MNPs), with polyurea nano-capsules (PU NCs) having an average size of 5–20 nm was carried out through interfacial polycondensation between amine and isocyanate monomers in inverse nano-emulsion (water-in-oil). The desired magnetic PU NCs were obtained utilizing toluene and triple-distilled water as continuous and dispersed phases respectively, polymeric non-ionic surfactant cetyl polyethyleneglycol/polypropyleneglycol-10/1 dimethicone (ABIL EM 90), diethylenetriamine, ethylenediamine diphenylmethane-4,4′-diisocyanate, and various percentages of the ionic liquid-modified MNPs. High loading of the ionic liquid-modified MNPs up to 11 wt% with respect to the dispersed aqueous phase was encapsulated. The magnetic PU NCs were probed using various analytical instruments including electron microscopy, infrared spectroscopy, X-ray diffraction, and nuclear magnetic spectroscopy. This unequivocally manifested the successful synthesis of core-shell polyurea nano-capsules even without utilizing osmotic pressure agents, and confirmed the presence of high loading of MNPs in the core.

## 1. Introduction

Nano-capsules, composed of liquid or hollow cores, enclosed in a nontoxic polymeric shell, have been widely investigated for the encapsulation of hydrophobic and hydrophilic substances [1]. Different methods for fabricating nano-capsules and nanoparticles have been developed. Typically, they can be synthesized through interfacial polymerization, suspension polymerization, and nanoprecipitation in oil-in-water (O/W), oil-in-oil (O/O), and water-in-oil (W/O) emulsions, as well as mini- and micro-emulsions [2,3,4,5,6,7,8]. Interfacial polymerization and polycondensation is one of the most studied methods for fabricating a wide range of functional polymeric nano-capsules. In this process, polymerization occurs at the interface between two immiscible phases, with each phase containing dissolved complementary monomers, resulting in nano-capsules and nanoparticles with sizes on the order of emulsion droplets [9,10]. 

To encapsulate hydrophilic compounds, an inverse nano-emulsion (W/O system) was utilized [11,12]. Inverse nano-emulsion, also known as inverse miniemulsion, consists of 50–500 nm surfactant-stabilized aqueous droplets dispersed in a hydrophobic organic continuous phase. Nano-emulsions are kinetically stable and their preparation requires high energy, for example, using high shear homogenization and ultra-sonication methods [13,14].

In order to obtain the desired polymeric nano-capsules, stable inverse nano-emulsions must be prepared. This is achieved by using a combination of non-ionic surfactants with low hydrophilic-lipophilic balance (HLB) and osmotic pressure agents (lipophobes) as co-stabilizers. The surfactant sterically stabilizes the droplets and lipophobes within the droplets, and prevents droplet coalescence by suppressing Ostwald ripening [15,16,17].

Among the nanostructured materials prepared via interfacial polymerization, polyurea has gained the most interest due to having properties such as biocompatibility, high mechanical characteristics, and surface functionality [14,18,19]. Only a few studies have reported the preparation of polyurea nano-capsules (PU NCs) by means of inverse miniemulsion by utilizing lipophobes. In this regard, Landfester and co-workers reported the preparation of hollow polyurea, polythiourea, as well as polyurethane nano-capsules and nanoparticles [20]. They studied the effect of monomers and solvents on the shell thickness and morphology to develop nanoreactors for preparing silver nanoparticles (NPs). In another study, PU NCs were stabilized with an amino-functionalized surfactant and the encapsulation efficiency was examined by a fluorescent dye [19].

Nano-capsules have a high surface area-to-volume ratio, a narrow size distribution, and high encapsulation efficiency. However, the isolation and recovery of such systems is difficult and requires time consuming and tedious procedures. Nevertheless, these efforts can be minimized by encapsulating magnetite nanoparticles (MNPs), which will endow the PU NCs with superparamagnetic properties. This process facilitates their isolation simply by applying an external magnetic field [21,22,23,24]. The polymeric shell protects the MNPs from undergoing agglomeration and oxidation, which otherwise leads to a loss of magnetic properties.

Previously, we reported the synthesis of magnetically separable PU nanoparticles formulated using O/O nano-emulsion by employing the interfacial polycondensation reaction between 2,6-diaminopyridine and polymethylene-polyphenyl isocyanate (PAPI 27) in the presence of poly(1-ethenylpyrrolidin-2-one/hexadec-1-ene) (Agrimer AL 22) surfactant [25]. Spherical particles of ~450 nm size were obtained. In line with that research, we proposed that magnetically separable PU NCs be prepared from inverse nano-emulsion. To the best of our knowledge, encapsulation of MNPs within PU NCs (MNPs@PU NCs), prepared from W/O nano-emulsions, has not been thoroughly investigated [19]. We believe that the encapsulated MNPs within polymeric capsules and matrixes, in which the attributes of PU and MNPs are combined, may be of great interest and can be applied in various fields such as biotechnology, medicine, catalysis, magnetic resonance imaging, agriculture, and other environmental and industrial applications [26,27,28,29]. Therefore, the aim of this work was to synthesize and provide an elaborative study as well as to thoroughly characterize new MNPs@PU NCs prepared in a facile manner from inverse nano-emulsion. In addition, we focused on studying the effect of different parameters, such as osmotic pressure agents, amine and isocyanate monomers, solvents, and surfactants.

Here, the magnetic polyurea nano-capsules were prepared through interfacial polycondensation in W/O nano-emulsion. The synthesis involved nano-emulsification of an aqueous phase containing ionic liquid (IL) stabilized magnetite nanoparticles, amine monomers, and an oil phase containing a polymeric non-ionic surfactant. This was followed by the addition of diisocyanate monomer to initiate the interfacial polycondensation, forming a polyurea shell and MNPs encapsulated within the core.

## 2. Results and Discussions

### 2.1. The Formation of Polyurea Nano-Capsules from Water-in-Oil (W/O) Nano-Emulsion

**Figure 1:**
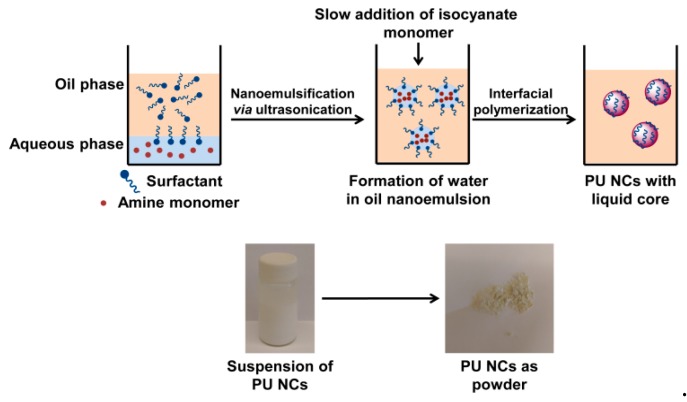
Preparation of polyurea nano-capsules from water-in-oil (W/O) nano-emulsion.

Prior to the encapsulation of the MNPs, an optimal composition for the synthesis of PU NCs was established through interfacial polymerization reactions in inverse nano-emulsions, as illustrated in Figure 1.

**Scheme 1:**
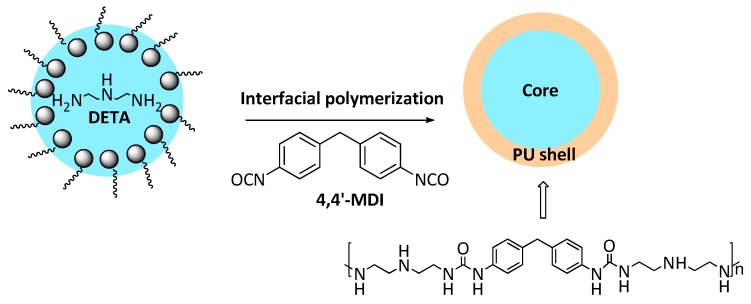
The formation of polyurea through interfacial polymerization between diethylene triamine (DETA) and diphenylmethane-4,4′-diisocyanat (4,4′-MDI).

The polyurea nano-capsules were produced in two steps. Briefly, in the first step, the aqueous phase, composed of triple-distilled water (TDW), amine monomer, and lipophobe, was nano-emulsified by homogenization, followed by ultrasonication in an oil phase consisting of a non-polar organic solvent and surfactant. Subsequently, diisocyanate monomer was slowly added to the nano-emulsion system while sonication and the interfacial polycondensation were initiated to form the PU shell. The reaction between the amine and isocyanate monomers is depicted in Scheme 1.

In order to attain stable water droplets dispersed in the oil phase, it is necessary to use the right surfactant. Thus, polymeric non-ionic surfactants with low HLB values were found to be most suitable, since they sterically stabilize the nanodroplets to prevent coalescence and provide a relatively condensed interface. Furthermore, an osmotic pressure agent, termed a lipophobe, was mainly used to maintain the stability of the nano-emulsion during the polymerization process [20]. This, hinders Ostwald ripening caused by nano-emulsion polydispersity and consequently, osmotic pressure forms inside the aqueous droplets, which reduces the Laplace pressure. Hence, it prevents the formation of aggregates.

In the above process, in order to obtain the optimal composition, various parameters in different proportions were tested, such as the type of surfactant, the amine and isocyanate monomers, the organic solvent, and the lipophobe in different ratios was also examined.

#### 2.1.1. Variation of the Type and Amount of Surfactant

In preliminary experiments, different percentages of various surfactants with low HLB values were investigated using inverse nano-emulsions consisting of 90 wt% toluene as the continuous phase and 10 wt% TDW as the dispersed phase, and diethylene triamine (DETA) and diphenylmethane-4,4′-diisocyanat (4,4′-MDI) as the amine and isocyanate monomers.

**Figure 2:**
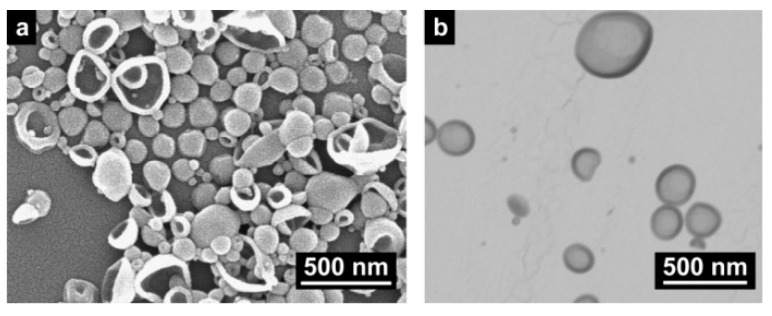
The morphology of PU NCs prepared using 1 wt% of ABIL EM 90 as the surfactant: (**a**) SEM and (**b**) STEM images.

Aggregates were formed after adding the diisocyanate monomer 4,4′-MDI to systems containing 1 wt% and 5 wt% of anionic surfactant (sodium 1,4-bis(2-ethylhexoxy)-1,4-dioxobutane-2-sulfonate) (AOT), non-ionic surfactants sorbitane monooleate (SPAN80), or polyoxyethylene (2) stearyl ether (Brij 72). Aggregates were also obtained when 1 wt% of amphipathic emulsifier lecithin was used, as confirmed by scanning electron microscopy (SEM) analysis (Figure S1a). However, aggregates with some nano-capsules having incomplete polymerization were obtained when 1 wt% nonionic polymeric surfactant Agrimer AL22 was employed (Figure S1b), whereas nano-capsules with core-shell morphology were obtained with 1 wt% cetyl polyethyleneglycol/polypropyleneglycol-10/1 dimethicone (ABIL EM 90) (PU-1, Table S1), as confirmed by SEM and scanning transmission electron microscopy (STEM) analysis (Figure 2).

The presence of the nano-capsules strongly indicates that polymeric surfactants are more compatible with the W/O system. This could be attributed to the fact that the polymeric surfactants produce several adsorption sites with negligible desorption from the emulsion interface. As shown by SEM, ABIL EM 90 provided better results than Agrimer AL22, indicating that ABIL EM 90 is more compatible in inverse nano-emulsion systems, presumably due to the enhancement of droplet stability and the strength of the interfacial film. Further optimizations, such as varying the surfactant percentage, the type and ratio of amine and isocyanate monomers, and varying the organic solvents, were carried out with ABIL EM 90 as the surfactant.

The stability of the W/O nano-emulsion as well as the resulting PU NCs depends not only on the type—it also depends on the amount of the applied surfactant. Therefore, different percentages (0.25%, 0.5%, 1%, 2%, 3%, 4%, and 5%) of ABIL EM 90 were employed. SEM analysis clearly indicated the formation of aggregates with less than 1% surfactant (Figure S2a,b). Interestingly, the size and polydispersity as well as the stability of the PU NCs were hardly affected by increasing the percentage of ABIL EM 90, however, at 3% and more, the PU NCs became more attached, as observed in SEM (Figure S2e–g). This shows that 1% (Figure S2c) and 2% (Figure S2d) of the surfactant afforded the best results. Because it is preferable to employ the minimum amount of surfactant, 1% ABIL EM 90 was chosen as the optimal condition and was used for additional optimization steps.

#### 2.1.2. Variation of the Type and Percentage of the Continuous Organic Phase

Further optimization was carried out by examining the effect of the organic solvent on the stability and morphology of the PU NCs while utilizing the same composition mentioned above with 1 wt% ABIL EM 90, DETA (2.9 mmol), and 4,4′-MDI (2.9 mmol). Aggregates were formed with cyclohexane as the continuous phase (Figure S3a). However, in the presence of heptane, a mixture of PU particles, aggregates, and NCs was obtained (Figure S3b), whereas xylene provided a result similar to toluene (Figure S3c). These diverse morphologies were obtained presumably due to solubility differences regarding the isocyanate monomer in the aliphatic and aromatic continuous organic phases. In this regard, 4,4′-MDI has better solubility in toluene and xylene, compared with heptane and cyclohexane, hence, aggregates were formed in the aliphatic solvents. An additional parameter affecting the morphology is related to interfacial tension between the aqueous and organic phases. ABIL EM 90, at the specific applied concentration, seems to sufficiently lower the interfacial tension between toluene and water, rather than between cyclohexane or heptane and water, thus enhancing the stability of the nano-emulsion droplets and forming the desired nano-capsules.

**Figure 3:**
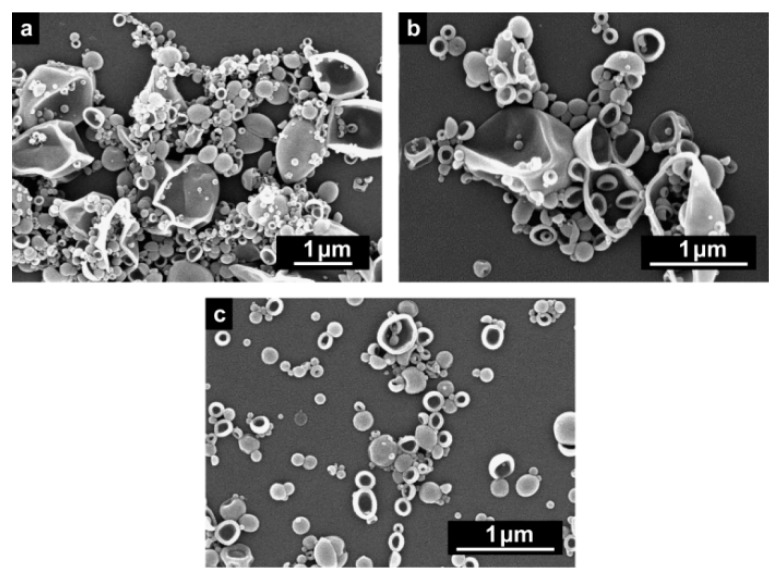
SEM images of PU NCs prepared using different continuous: aqueous phase ratios: (**a**) 70%:30%, (**b**) 80%:20 %, and (**c**) 85 %:15 %.

To further examine the influence of the continuous phase on the formation and size distribution of the PU NCs, different percentages of a continuous phase (70%, 80% 85%, and 90%) consisting of toluene and ABIL EM 90 (1 wt%) were investigated. SEM analysis revealed the formation of polydispersed PU NCs systems with all percentages tested (Figure 3).

**Figure 4:**
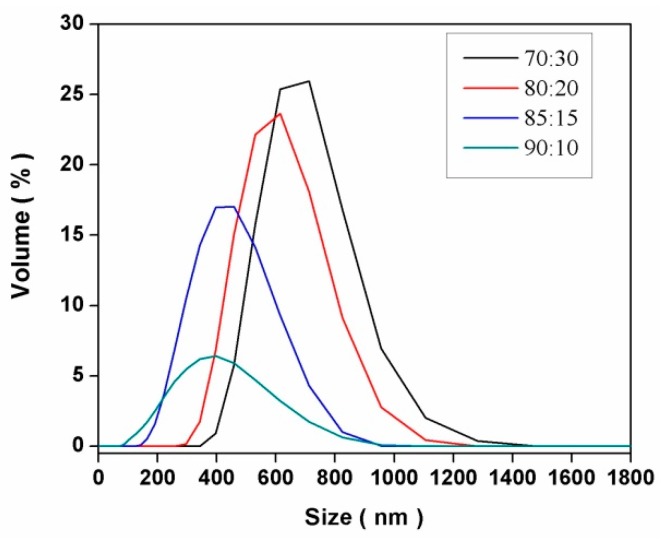
The influence of continuous: dispersed phase ratios of 70%:30%, 80%:20%, 85%:15%, and 90%:10% on the PU NCs size.

In addition, dynamic light scattering (DLS) studies revealed an increase in the average PU NCs size, along with a decrease in the percentage of the continuous phase and a simultaneous increase in the amount of the aqueous phase. When 70%, 80%, 85%, and 90% of the continuous phase were used, average sizes of 897 nm, 655 nm, 242 nm, and 270 nm were obtained, respectively (Figure 4).

#### 2.1.3. Variation of the Type of the Polyurea (PU) Monomers

Initially, DETA and 4,4′-MDI were utilized as the amine and isocyanate monomers. An additional optimization step was conducted by varying the type and ratio of the amine and isocyanate monomers utilizing 1% of ABIL EM 90 and 10% of the aqueous phase containing poly(acrylamide-*co*-diallyldimethylammonium chloride (polyquaternium 7) as the lipophobe. This process appears to be essential, since the shell thickness and flexibility as well as the porosity and permeability of the PU NCs play a key role in the effectiveness and applicability of the system. Therefore, various amine monomers such DETA, ethylenediamine (EDA), 1,6-hexamethylenediamine (HMDA), and isocyanate monomers such as (PAPI 27, 4,4′-MDI, toluenediisocyanate (TDI), 1,6-hexamethylene diisocyanate (HDI), and 4,4′-methylenebis(cyclohexy isocyanate) (HMDI) were tested.

**Figure 5:**
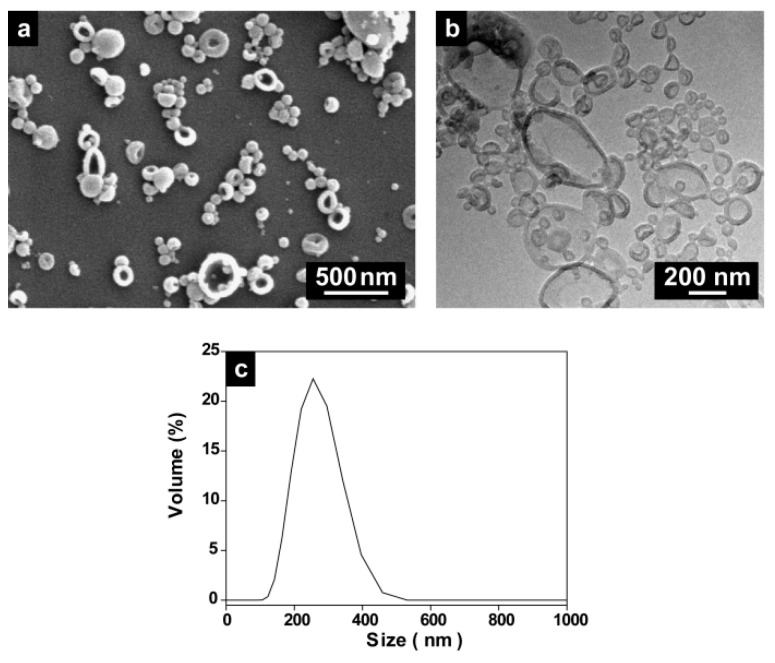
(**a**) SEM, (**b**) TEM, and (**c**) the size distribution of PU NCs prepared using 4,4′-MDI and a mixture of DETA and EDA as the monomers (PU-9).

The combination of EDA with HMDI (PU-6), PAPI 27 (PU-5), 4,4′-MDI (PU-8), or TDI (PU-7), as well as DETA with TDI (PU-4), PAPI 27 (PU-2), or HMDI (PU-3) (Table S1) afforded either aggregates or an incomplete formation of the PU shell, as observed in SEM (Figure S4). Polydispersed PU NCs with an average size of 259 nm, as revealed by SEM, transmission electron microscopy (TEM), and DLS analyses (Figure 5), were obtained when 4,4′-MDI and a mixture of DETA and EDA were utilized as the PU monomers (PU-9, Table S1).

#### 2.1.4. The Influence of the Electrolyte

**Figure 6:**
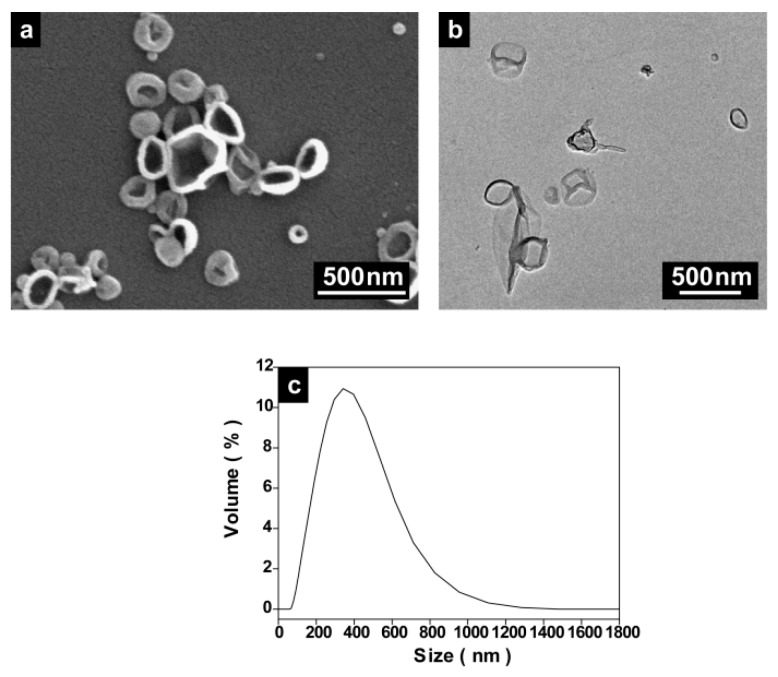
(**a**) SEM, (**b**) TEM, and (**c**) the size distribution of PU-15 prepared using 80%:20% of the continuous and dispersed phases, respectively.

In order to suppress Ostwald ripening and obtain stable and non-aggregated capsules, an electrolyte is usually added to the aqueous phase. The influence of the electrolyte polyquaternium 7 on the size and morphology of the PU NCs was further studied using the PU-1 system. Varying the percentage of polyquaternium 7, namely, 0% (PU-10), 1% (PU-11), 2% (PU-12), and 3% (PU-13) (Table S1) had no appreciable effect on the morphology of PU NCs, as observed in SEM (Figure S5a–d). However, with 1% NaCl (PU-14, Table S1), aggregates and flattened capsules were obtained (Figure S5e). Additionally, DLS measurements revealed that the size distribution of PU-10 and PU-13 (Figure S6a,b) were similar to PU-1. These analyses clearly show that PU NCs are not affected by the electrolytes and can be formed even without utilizing them. An additional system with 80%:20% of continuous and aqueous phases respectively, without electrolyte was prepared (PU-15, Table S1). In this case, PU NCs with a core shell structure were also formed, as confirmed by SEM and TEM (Figure 6a,b. DLS analysis, in agreement with SEM, revealed a polydispersed system with an average nano-capsule size of 365 nm (Figure 6c).

**Figure 7:**
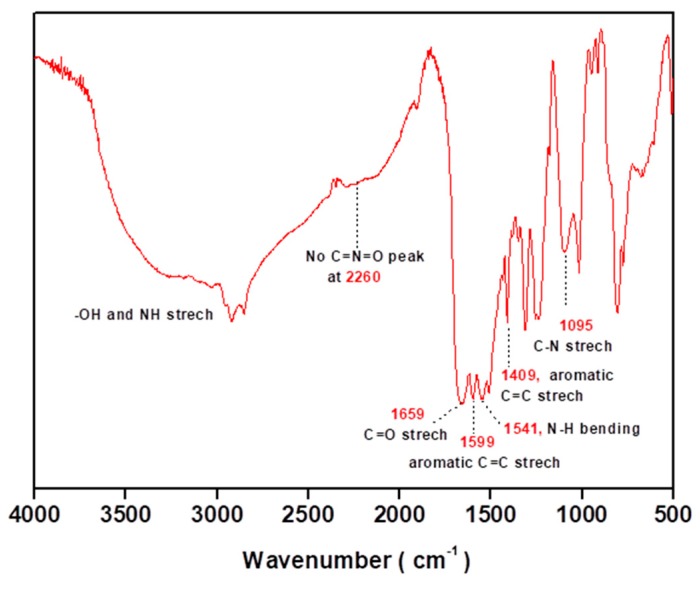
The FTIR spectrum of PU-15.

The complete polymerization of the amine with the isocyanate monomers was revealed by Fourier transform infrared (FTIR) analysis (Figure 7). The presence of absorbance peaks above 3000 cm^−1^, which correspond to the overlapping stretching vibrations of the N–H and –OH (from water) groups, and an absorption band at 1659 cm^−1^ assigned to C=O stretching vibrations, indicate the formation of polyurea [18]. In addition, the absence of the absorbance peak of the isocyanate group (–C=N=O) at 2260 cm^−1^ indicates the complete consumption of the isocyanate monomer. Furthermore, absorbance peaks at 1599 cm^−1^ and 1409 cm^−1^ can be attributed to C=C stretching vibrations of aromatic rings. The absorbance bands at 2852 and 2922 cm^−1^ are ascribed to sp3-hybridized C–H, and the peak at 3028 cm^−1^ is attributed to sp2 C–H stretching vibrations. In addition, absorbance bands at 1541 and 1095 cm^−1^ are ascribed to the secondary N–H bending and the C–N stretching vibrations, respectively.

**Figure 8:**
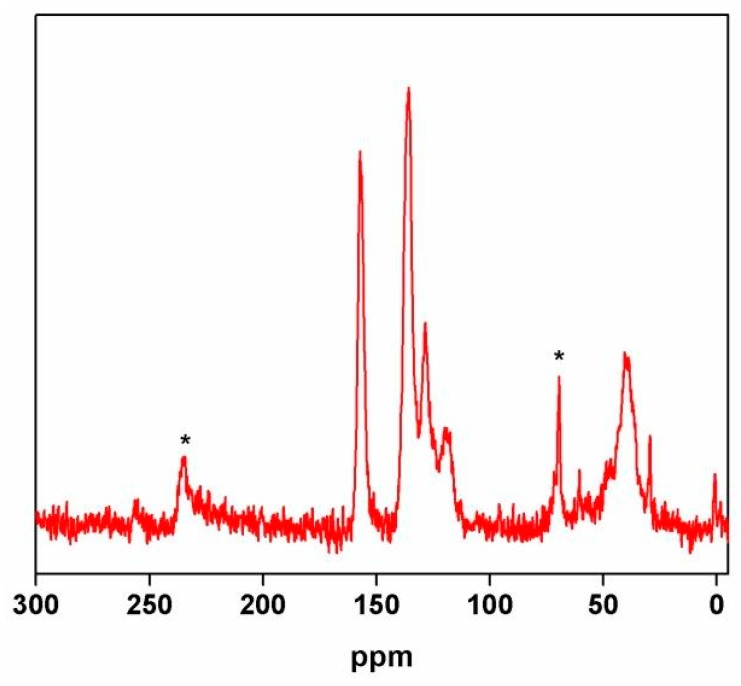
The ^13^C CP-MAS NMR of PU NCs (PU-15); * indicates the spinning sideband.

The formation of the PU NCs (PU-15) was further confirmed by solid state carbon nuclear magnetic resonance (^13^C CP-MAS NMR) (Figure 8). Peaks at 21–48 ppm and 115–135 ppm are attributed to the aliphatic (from the DETA and EDA monomer) and aromatic (from 4,4′-MDI monomer) carbons of the PU shell. The peak at 156 ppm is ascribed to the carbonyl group (C=O) of the urea, indicating the formation of polyurea [18,30].

### 2.2. Encapsulation of Ionic Liquid-Modified Magnetite Nanoparticles (MNPs-IL-C_4_@PU NCs)

Magnetic polymer nanomaterials that combine the properties of organic and inorganic components have been fabricated for various applications in biomedical, environmental, sensing, drug delivery, and magnetic resonance imaging (MRI) [31,32,33,34,35,36].

**Figure 9:**
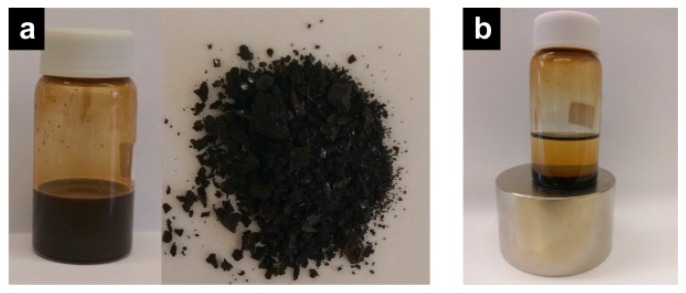
(**a**) MNPs-IL-C_4_@PU NCs as suspension and powder and (**b**) a magnetic separation of MNPs-IL-C_4_@PU NCs.

Magnetic nanoparticles have also been applied in catalysis to facilitate the isolation and recovery of the nano-catalysts and catalyst nano-supports by applying an external magnetic field. Herein, we have prepared magnetic PU NCs in an inverse nano-emulsion (Figure 9a), which could have great potential in the above-mentioned fields.

Incorporating MNPs within the PU NCs would impart superparamagnetic properties to the NCs, enabling them to be suitable for various applications such as magnetic nano-catalysts, which can be conveniently recovered under an external magnetic field (Figure 9b). We encapsulated different percentages (1–11 wt% with respect to the aqueous phase) of pre-prepared and stabilized MNPs with ionic liquid-based silane, 1-butyl-3-(3-(trimethoxysilyl)propyl)-1H- imidazol-3- chloride (IL-C_4_) [25], (MNPs-IL-C_4_) with an average size of 5–20 nm in the core of PU NCs using a composition employed for preparing the PU-15 system (Table 1).

**Figure 10:**
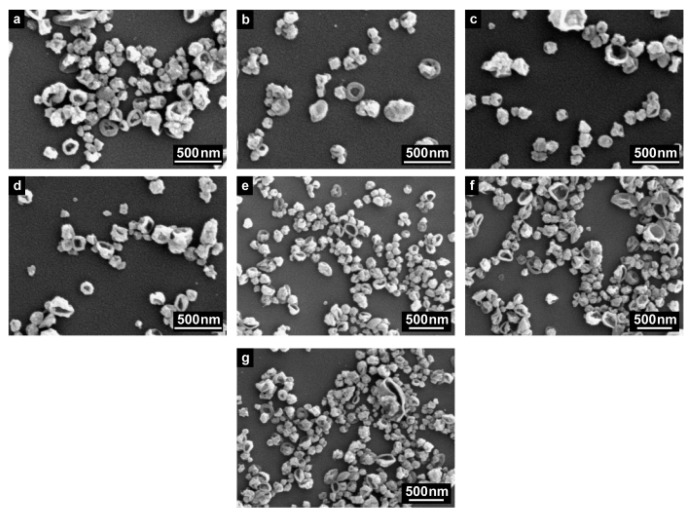
SEM images of MNPs-IL-C_4_@PU NCs. (**a**) 1% (PU-15a), (**b**) 2% (PU-15b), (**c**) 3% (PU-15c), (**d**) 5% (PU-15d), (**e**) 7% (PU-15e), (**f**) 9% (PU-15f), and (**g**) 11% (PU-15g) of encapsulated MNPs-IL-C_4_.

**Figure 11:**
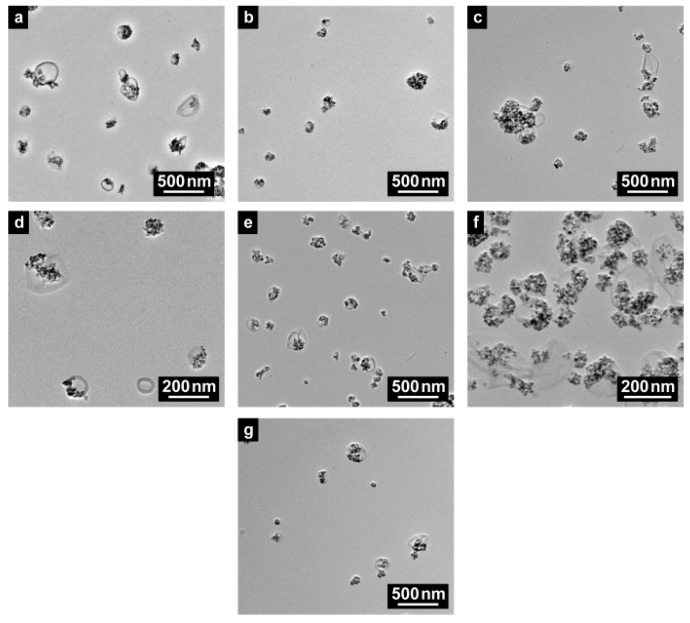
TEM images of MNPs-IL-C_4_@PU NCs. (**a**) 1% (PU-15a), (**b**) 2% (PU-15b), (**c**) 3% (PU-15c), (**d**) 5% (PU-15d), (**e**) 7% (PU-15e), (**f**) 9% (PU-15f), and (**g**) 11% (PU-15g) of encapsulated MNPs-IL-C_4_.

The surface morphology of PU-15a-15g was probed by SEM (Figure 10) and TEM (Figure 11) analyses, which confirmed the formation of PU NCs with MNPs-IL-C_4_ encapsulated in the core of the NCs. Since MNPs-IL-C_4_ is hydrophilic, it does not dissolve in the non-polar continuous phase, therefore, non-encapsulated MNPs were not observed. Additionally, the size distribution of MNPs-IL-C_4_@PU NCs decreased, compared with pure PU NCs, however, increasing the amount of MNPs had little effect on the polydispersity and average size distribution (Table 1, Figure S7). Moreover, FTIR analysis of PU-15a-15g exhibited similar results as pure PU NCs (PU-15), confirming the formation of polyurea shell (Figure S8).

**Figure 12:**
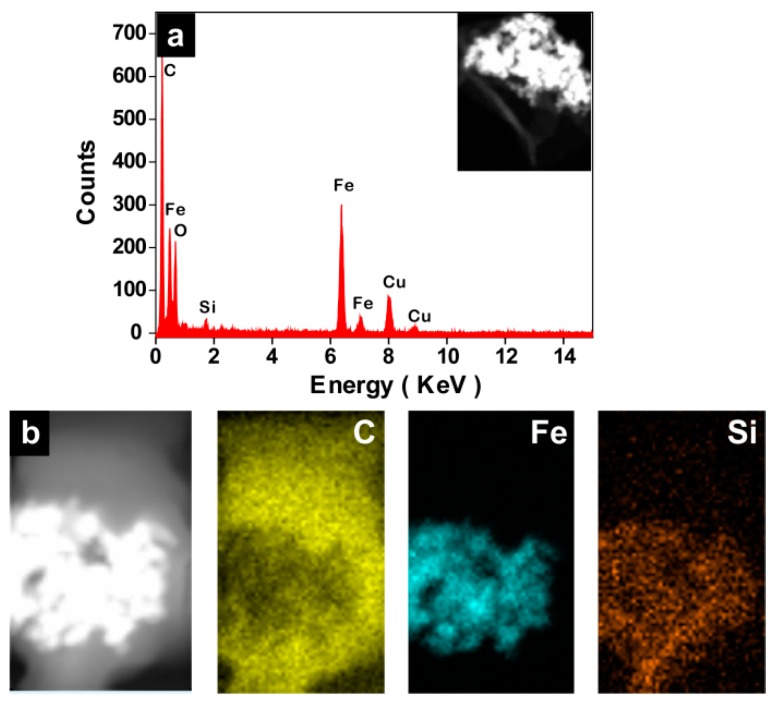
(**a**) STEM/ EDS and (**b**) EDS mapping analyses of MNPs-IL-C_4_@PU NCs (PU-15b).

STEM/energy dispersive X-ray spectroscopy (EDS) and element mapping analyses were carried out in order to obtain more information about the composition and structure of the MNPs-IL-C_4_@PU NCs. STEM/EDS analysis (Figure 12a) also confirmed the presence of an iron element content of the MNPs in the core of the PU NCs and showed the existence of an Si element attributed to the silane group of IL-C_4_ supported on the MNPs and of ABIL EM 90.

The elemental distribution of MNPs-IL-C_4_@PU NCs was further probed by EDS mapping analysis (Figure 12b). In agreement with TEM, EDS mapping displayed the distribution of iron (Fe), indicating that the MNPs are located in the core of the PU NCs (Figure 12b, blue map). When comparing the distribution zones of C with the Si and Fe elements, it can be clearly seen that the C element (Figure 12b, yellow map) of the PU skeleton is distributed throughout all the areas of the NCs, whereas the Si element (Figure 12b, orange map), which is a component of silane IL-C_4_ and ABIL EM 90, is more localized in the center.

**Figure 13:**
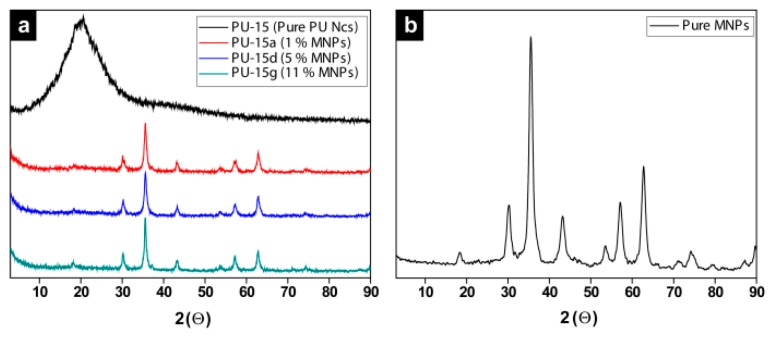
XRD pattern of (**a**) PU NCs (PU-15) and MNPs-IL-C_4_@PU NCs and (**b**) pure MNPs.

The composition of the PU NCs, pure MNPs, and MNPs-IL-C_4_@PU NCs was also probed by X-ray powder diffraction (XRD). The XRD pattern of PU NCs (PU-15) displayed a broad peak in the range 2θ = 10–30°, which is attributed to the amorphous polyurea. The XRD of PU-15a, PU-15d, and PU-15g revealed the presence of amorphous polyurea and showed the characteristic peaks of MNPs at 2θ = 18.1°, 30.2°, 35.6°, 43.2°, 53.6°, 57.2°, and 62.8° (Figure 13).

**Figure 14:**
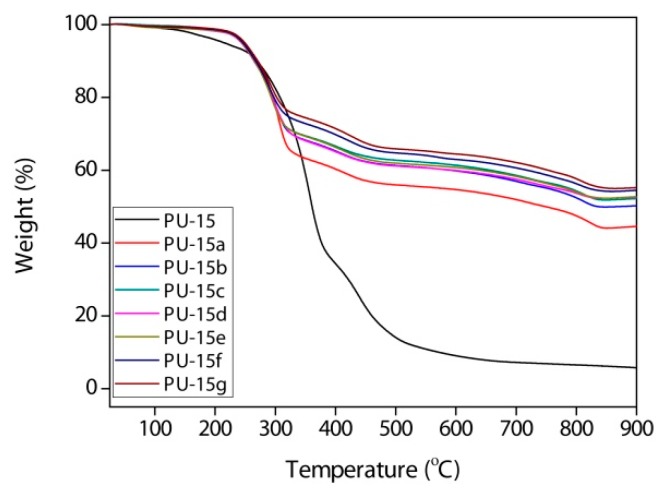
TGA analysis of pure PU NCs (PU-15) and MNPs-IL-C_4_@PU NCs (1–11%).

The thermogravimetric analysis (TGA) of pure PU NCs and MNPs-IL-C_4_@PU NCs (1–11%) over a temperature range of 25–950 °C under an inert atmosphere and at a heating rate of 10 °C/min revealed that pure PU NCs (PU-15) have two degradation steps with a total 94.7% weight loss (Figure 14). The first decomposition step was observed at 125–390 °C, which is attributed to the initial decomposition of the PU shell, toluene, and water [30,37]. The second step, which was at a temperature higher than 390 °C, is attributed to the additional decomposition of PU. The TGA curves of MNPs-IL-C_4_@PU NCs revealed that when the 1, 2, 3, 5, 7, 9, or 11 wt% of MNPs per aqueous phase was added during the encapsulation process, the measured weight percentage of MNPs was 45.02%, 50.59%, 52.63%, 52.79%, 53.03%, 54.85%, or 55.64%, respectively. Moreover, TGA gives an indication of the stability of the PU NCs. It was clearly seen that all MNPs-IL-C_4_@PU NCs systems exhibited three degradation steps with the initial decomposition temperature at ~220 °C. This indicates that the presence of MNPs-IL-C_4_ increased the thermal stability of the PU NCs. The degradation steps at 220–500 °C and >500 °C are attributed to the decomposition of the PU shell and the IL group attached. Theoretically, pure PU should exhibit a 100% weight loss, since it is composed of pure organic material. However, PU-15 revealed the presence of 5.3% of non-decomposable material, which could be attributed to the inorganic surfactant ABIL-EM 90 and some species formed during the heating process.

## 3. Materials and Methods

Cetyl polyethyleneglycol/polypropyleneglycol-10/1 di-methicone (ABIL EM 90) was denoted by Sol-Gel Technologies (Ness Ziona, Israel); poly(1-ethenylpyrrolidin-2-one/hexadec-1-ene) (Agrimer AL 22) and polymethylene-polyphenyl isocyanate (PAPI 27) were contributed by FMC Corporation (Ewing, NJ, USA). FeCl_3_ × 6H_2_O, FeCl_2_ × 4H_2_O, and ammonium hydroxide 25% were purchased from Acros Fischer Scientific through their distributor in Israel, Holland Moran LTD (Yehud, Israel). (3-chloropropyl) trimethoxysilane and 1-butyl imidazole were purchased from Sigma Aldrich (Rehovot, Israel). All amine and isocyanate monomers were purchased from Sigma-Aldrich or Acros Fischer.

Scanning electron microscopy (SEM) was utilized to determine the morphology of the PU NCs. The SEM analyses were carried out using a high-resolution scanning electron microscope (HR SEM) Sirion (FEI Company, Hillsboro, OR, USA) using a Schottky-type field emission source and a secondary electron detector. The images were scanned at a voltage of 5 kV. Transmission electron microscopy (TEM) and scanning transmission electron microscopy/energy dispersive X-ray spectroscopy (STEM/EDS) were performed with (S) TEM Tecnai F20 G2 (FEI Company, Hillsboro, OR, USA) operated at 200 kV. The Fourier transform infrared spectra (FTIR) were recorded at room temperature in transmission mode using a Perkin Elmer spectrometer 65 FTIR instrument (Waltham, MA, USA). Thermogravimetric analysis (TGA) was performed on a Mettler Toledo TG 50 analyzer (Greifensee, Switzerland). Measurements were carried out over a temperature range of 25–950 °C, at a heating rate of 10 °C/min under nitrogen. Dynamic light scattering (DLS) was utilized to determine the size distribution of the PU-NCs. These measurements were performed on a Nano Series instrument of model Nano-Zeta Sizer (Malvern Instruments, Worcestershire, United Kingdom) model ZEN3600. Powder X-ray diffraction (XRD) measurements were performed on a D8 Advance diffractometer (Bruker AXS, Karlsruhe, Germany) with a goniometer radius of 217.5 mm, a secondary graphite monochromator, with 2° Sollers slits, and a 0.2 mm receiving slit. Low-background quartz sample holders were carefully filled with the powder samples. XRD patterns within the range 2θ = 1° to 90° were recorded at room temperature using CuKα radiation (λ = 1.5418 Å) with the following measurement conditions: a tube voltage of 40 kV, a tube current of 40 mA, step-scan mode with a step size of 2θ = 0.02°, and a counting time of 1 s/step. A solid-state ^13^C NMR spectrum was recorded with a Bruker DRX-500 instrument (Rheinstetten, Germany).

### 3.1. Synthesis of 1-Butyl-3-(3-(Trimethoxysilyl)Propyl)-1H-Imidazol-3-Cholride (IL-C_4_)

IL-C_4_ was synthesized using procedures reported previously [25]. Briefly, 3-chloropropyltrimethoxysilane (14.2 g, 114.3 mmol) and 1-butylimidazole (22.73 g, 114.3 mmol) were stirred under an inert atmosphere at 120 °C for 24 h. The mixture was cooled to room temperature to obtain a yellow-orange viscous liquid (35.32 g, 96% yield).

### 3.2. Preparation of Magnetite Nanoparticle-Supported IL-C_4_ (MNPs-IL-C_4_)

MNPs-IL-C_4_ was synthesized according to the procedure reported earlier [25].

### 3.3. Preparation of Polyurea Nano-Capsules (PU NCs)

The PU NCs were prepared in a typical procedure through interfacial polymerization in W/O nano-emulsion. The optimal PU NCs were prepared as follows: the continuous phase (organic phase, 40 g, 80%) consisted of toluene (37.5 g) and ABIL EM 90 (2.5 g, 5 wt%) was homogenized at 10,000 rpm for 30 s. A dispersed phase (aqueous phase, 10 g, 20%) consisted of triple-distilled water (TDW, 9.53 g), diethylene triamine (DETA, 2.9 mmol), and ethylenediamine (EDA, 2.9 mmol), which were then rapidly added during homogenization. The emulsification process was carried out for a further 1.5 min at 10,000 rpm, followed by sonication for 10 min using an ultrasonic cell disrupter with an output of 130 Watt and 20 KHz. Eventually, diphenylmethane-4,4′-diisocyanate (4,4′-MDI, 5.83 mmol), dissolved in 10 g total toluene, was slowly added to the nano-emulsion system during sonication. The mixture was then stirred for 3 h at room temperature. The resulting PU NCs were collected by centrifugation at 11,000 rpm for 15 min, washed two times with toluene, and finally re-dispersed in toluene to reach a 10 g suspension total.

### 3.4. Preparation of Magnetic PU NCs (MNPs-IL-C_4_@PU NCs)

Magnetic PU NCs were prepared by encapsulation of MNPs-IL-C_4_ in the PU NCs. The desired percentages of MNPs-IL-C_4_ were dispersed in the aqueous phase and sonicated until all MNPs were fully dispersed. The encapsulation process was achieved by following the same procedure and using the same amount of surfactant and components as described in the preparation of PU NCs. Finally, the reaction mixture was mechanically stirred at room temperature for 3 h. The resulting magnetic PU NCs were separated by an external magnetic field and re-dispersed in toluene.

## 4. Conclusions

PU NCs with magnetite nanoparticles encapsulated within the aqueous core were prepared via interfacial polycondensation in inverse nano-emulsion.

Various parameters such as surfactant, solvents, monomers, and lipophobes were thoroughly examined in different ratios and compositions. The obtained systems were characterized for their morphology, chemical composition, thermal stability, size, and encapsulation efficiency. Among the examined parameters and conditions, it was found that the polymeric non-ionic surfactant with a low HLB value, ABIL EM 90, proved to be the best stabilizer for the inverse nano-emulsion and hence, for the established NCs. The desired PU NCs were obtained with DETA, EDA, and 4,4′-MDI as the PU monomers.

The morphology and size distribution of PU NCs were not affected by increasing the percentage of polyquaternium 7, indicating that the NCs can be formulated even without employing electrolytes as osmotic pressure agents. The encapsulation efficiency of PU NCs was examined by encapsulating up to 11% of MNPs-IL-C_4_. The presence of the MNPs, regardless of the percentage, resulted in increased thermal stability of the PU NCs, as confirmed by TGA analysis. SEM and TEM analyses confirmed that the MNPs-IL-C_4_ were not adsorbed on the capsules’ shell but rather, were encapsulated in the aqueous core. Nonetheless, the average size distribution of the magnetic PU NCs decreased when compared with pure PU NCs. This could be due to the imidazolium group on the MNPs causing a reduction of Ostwald ripening. Owing to the facile synthesis and biocompatibility of PU, the magnetic properties of MNPs, and the expeditious magnetic separation of the system, the proposed magnetic PU NCs systems may be utilized in various applications such as catalysis, targeted delivery of hydrophilic drugs, and in other biomedical applications both in academia and industry.

## Figures and Tables

**Table 1:** Composition and encapsulation of MNPs-IL-C_4_ in PU-15 ^a^.

Entry	Amine Monomer	Isocyanate Monomer	MNPs-IL-C_4_	Size (nm) ^b^
PU-15	DETA/EDA	4,4′-MDI	0%	365.5
PU-15a	DETA/EDA	4,4′-MDI	1%	233.7
PU-15b	DETA/EDA	4,4′-MDI	2%	216.4
PU-15c	DETA/EDA	4,4′-MDI	3%	221.8
PU-15d	DETA/EDA	4,4′-MDI	5%	195.1
PU-15e	DETA/EDA	4,4′-MDI	7%	198.5
PU-15f	DETA/EDA	4,4′-MDI	9%	246.8
PU-15g	DETA/EDA	4,4′-MDI	11%	238.7

^a^ Continuous phase (40 g, 80%) consisting of toluene (37.5 g) and ABIL EM 90 (2.5 g, 5 wt%). Dispersed phase (10 g, 20%) consisting of TDW (9.53 g), DETA (2.9 mmol), and EDA (2.9 mmol). Isocyanate (5.83 mmol) was dissolved in 10 g of total toluene and slowly added to the nano-emulsion system. ^b^ The average size was measured by dynamic light scattering (DLS).

## Supplementary Materials

The following are available online. Figure S1–S5: SEM images, Table S1: System composition for preparing PU NCs, Figure S6–S7: Size distribution of pure PU NCs and MNPs-IL-C_4_@PU NCs, Figure S8: FTIR spectra.
