# Supplementary material for: Magnetic Polyurea Nano-Capsules Synthesized via Interfacial Polymerization in Inverse Nano-Emulsion
Source: Molecules. 2019 Jul 23;24(14):2663. doi: 10.3390/molecules24142663 (PMC6680913; doi:10.3390/molecules24142663)
Supplement: Supplementary file 1 [file molecules-24-02663-s001.pdf]

Supporting Information

Magnetic Polyurea Nano-Capsules Synthesized via Interfacial Polymerization in Inverse Nano-Emulsion

Suzana Natour [1], Anat Levi-Zada [2] and Raed Abu-Reziq [1,*]

[1] Institute of Chemistry, Casali Centre of Applied Chemistry and Centre for Nanoscience and Nanotechnology, The Hebrew University of Jerusalem, Jerusalem 9190401, Israel
[2] Department of Entomology-Chemistry, Agricultural Research Organization, Volcani Centre, Rishon Lezion 7505101, Israel
* Correspondence: Raed.Abu-Reziq@mail.huji.ac.il; Tel.: +972-2-6586097

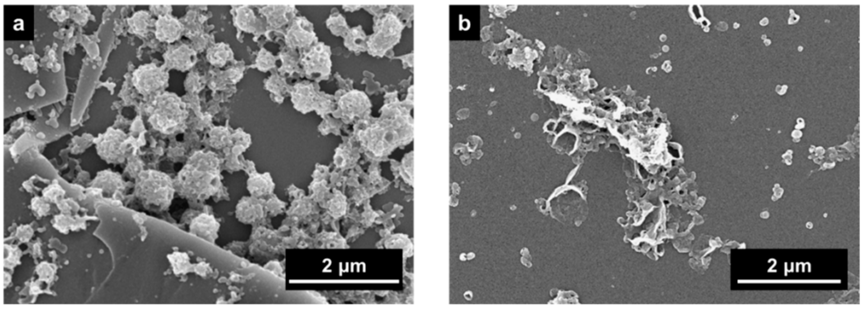

Figure 1. Effect of different surfactants on the formation of PU nanocapsules from W/O nanoemulsion a) Lecithin (1 wt%) and b) Agrimer AL22 (1 wt%).

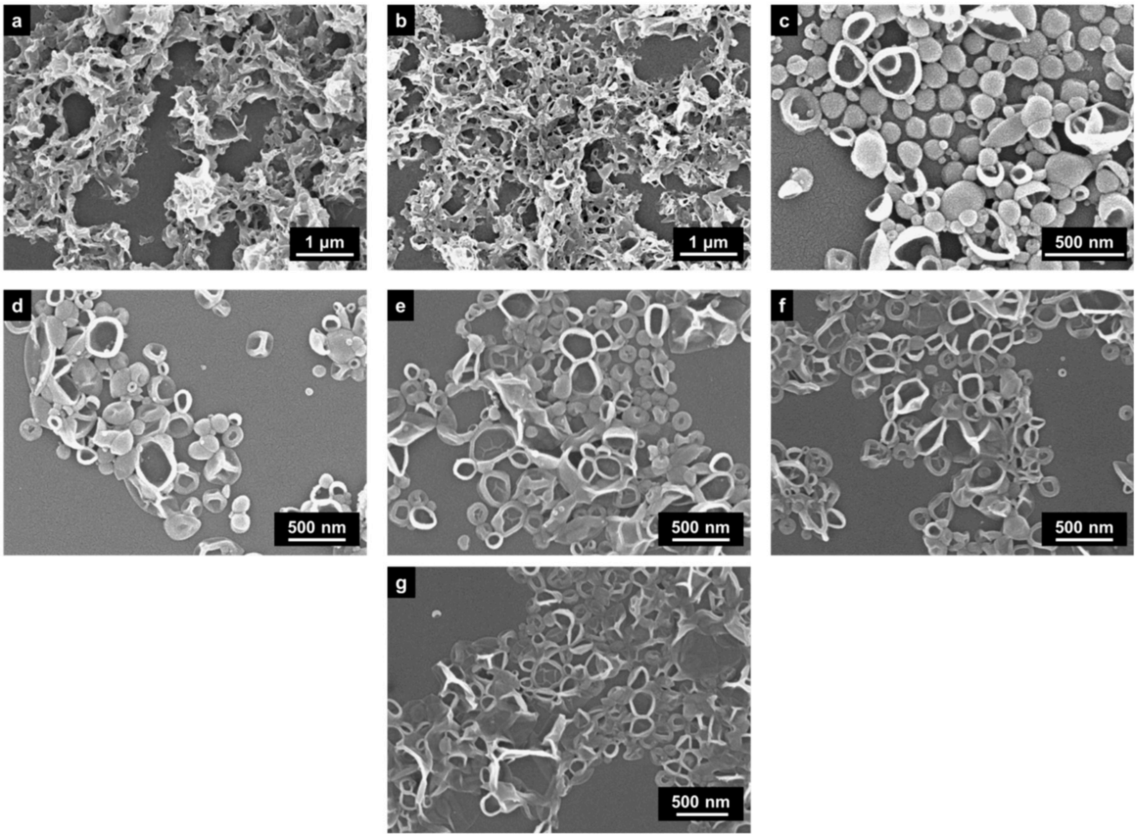

Figure S2. Effect of ABIL EM90 percentage on the formation of PU nanocapsules from W (10 wt%)/O (90 wt%) nanoemulsion. a) 0.25%, b) 0.5%, c) 1%, d) 2%, e) 3%, f) 4% and g) 5%.

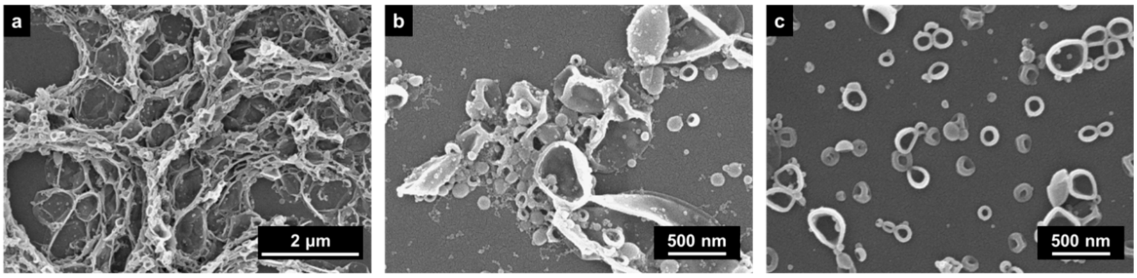

Figure S3. Effect of organic solvent as the continuous phase on the formation of PU NCs. a) Cyclohexane, b) heptane and c) xylene.

Table S1. System composition for the preparation of PU NCs.[a]

Entry	Amine monomer	Isocyanate monomer	Electrolyte
PU-1	DETA	4,4′-MDI	POLYQUATERNIUM 7 (0.25g, 5%)
PU-2	DETA	PAPI 27	POLYQUATERNIUM 7 (0.25g, 5%)
PU-3	DETA	HMDI	POLYQUATERNIUM 7 (0.25g, 5%)
PU-4	DETA	TDI	POLYQUATERNIUM 7 (0.25g, 5%)
PU-5	EDA	PAPI 27	POLYQUATERNIUM 7 (0.25g, 5%)
PU-6	EDA	HMDI	POLYQUATERNIUM 7 (0.25g, 5%)
PU-7	EDA	TDI	POLYQUATERNIUM 7 (0.25g, 5%)
PU-8	EDA	4,4′-MDI	POLYQUATERNIUM 7 (0.25g, 5%)
PU-9[b]	DETA / EDA	4,4′-MDI	POLYQUATERNIUM 7 (0.1g, 2%)
PU-10	DETA	4,4′-MDI	POLYQUATERNIUM 7 (0%)
PU-11	DETA	4,4′-MDI	POLYQUATERNIUM 7 (0.05g, 1%)

PU-12	DETA	4,4′-MDI	POLYQUATERNIUM 7 (0.1g, 2%)
PU-13	DETA	4,4′-MDI	POLYQUATERNIUM 7 (0.15g. 3%)
PU-14	DETA	4,4′-MDI	NaCl (0.05g, 1%)
PU-15[c]	DETA / EDA	4,4′-MDI	0%

[a] System composition: Continuous phase (45g, 90 %) comprised of toluene (44.5g) and ABIL EM90 (0.5g, 1 wt%). Dispersed phase (5g, 10%) comprised of TDW, POLYQUATERNIUM 7 (0.25g, 5 % per phase), amine monomer (2.9 mmol). Isocyanate (2.9 mmol) dissolved in 10g total toluene and slowly added to the nanoemulsion system. [b] DETA (1.45 mmol), EDA (1.33 mmol) and POLYQUATERNIUM 7 (0.1g, 2%) were used. [c] Continuous phase (40g,80 %) comprised of toluene (37.5g) and ABIL EM90 (2.5g, 5 wt%). Dispersed phase (10g, 20%) comprised of TDW (9.53g), DETA (2.9 mmol), EDA (2.9 mmol). Isocyanate (5.83 mmol) was dissolved in 10g total toluene and slowly added to the nanoemulsion system.

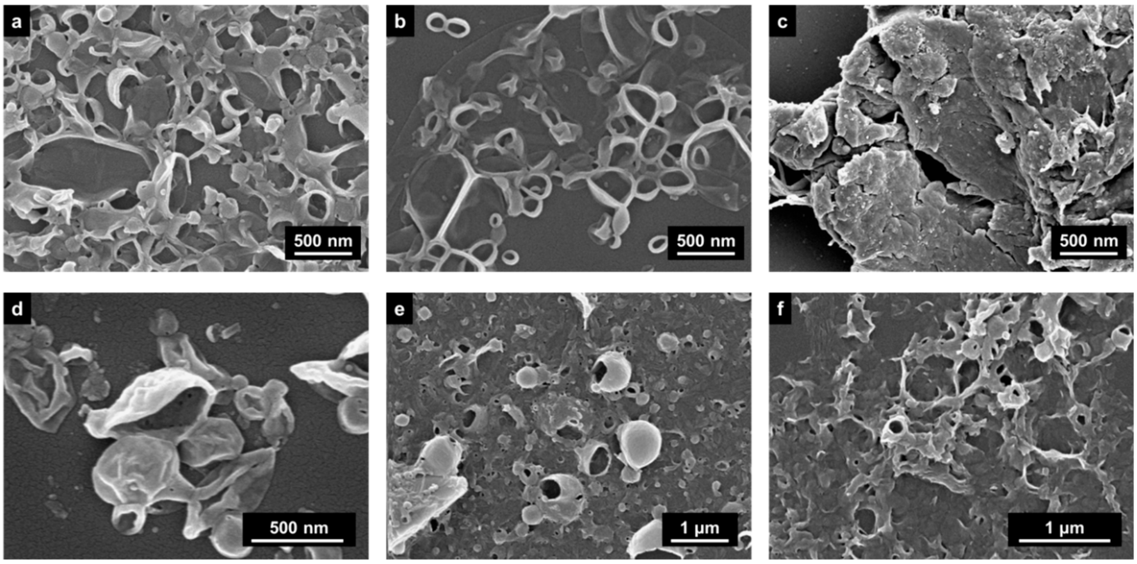

Figure S4. Effect of amine and isocyanate monomers on the formation of PU NCs. a) DETA: PAPI 27 (PU-2), b) DETA : HMDI (PU-3), c) DETA : TDI (PU-4), d) EDA : HMDI (PU-6), e) EDA: TDI(PU-7) and f) EDA : 4,4′-MDI (PU-8).

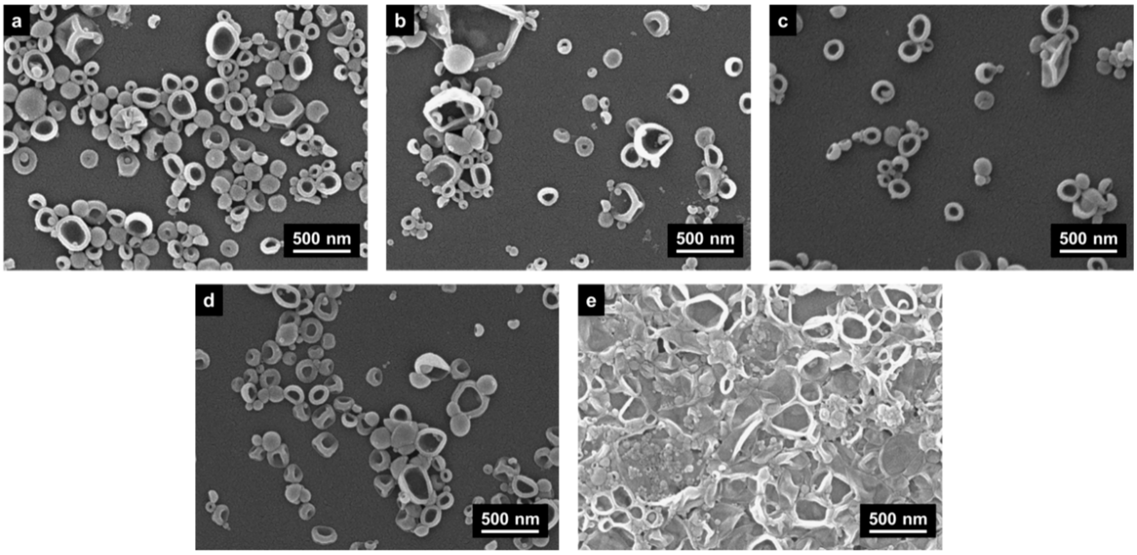

Figure S5. SEM images of PU NCs prepared using: a) 0% (PU-10), b) 1% (PU-11), c) 2% (PU-12) and d) 3% (PU-13) of POLYQUATERNIUM 7, and e) 1% NaCl (PU-14).

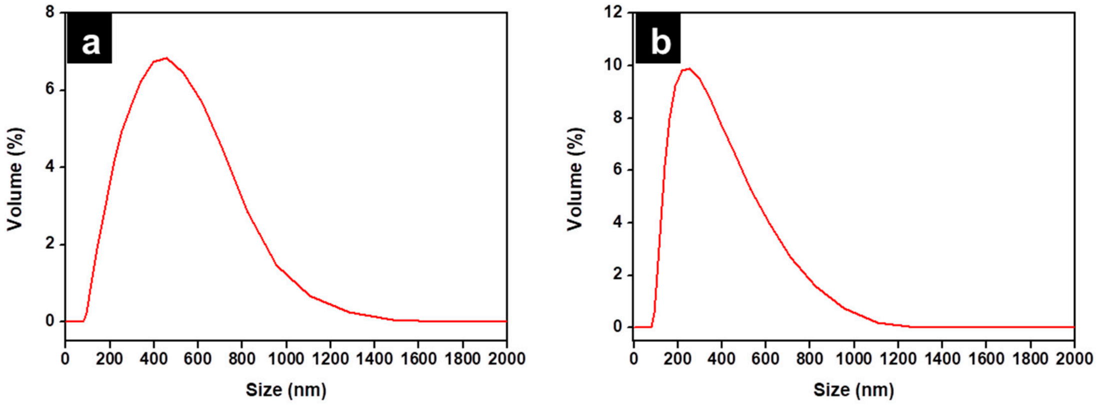

Figure S6 Size distribution of a) PU-10 and b) PU-13.

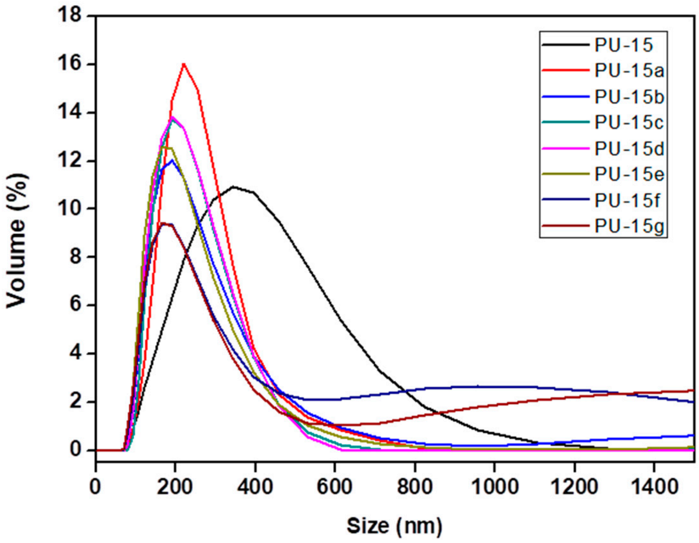

Figure S7. Size distribution of MNPs-IL-C_4@PU NCs.

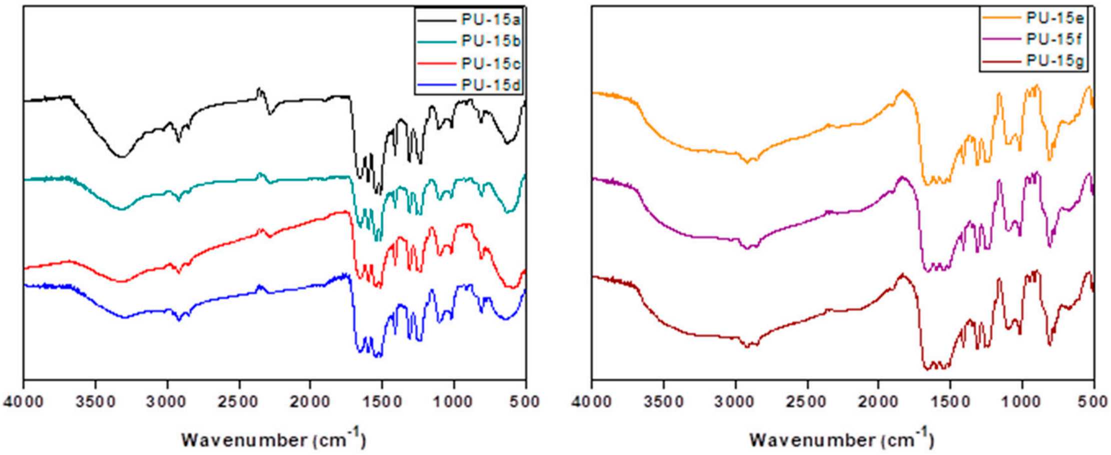

Figure S8. Transmission FTIR spectra of MNPs-IL-C_4@PU NCs.
